# DOCK4 Is a Platinum-Chemosensitive and Prognostic-Related Biomarker in Ovarian Cancer

**DOI:** 10.1155/2021/6629842

**Published:** 2021-02-03

**Authors:** Qianqian Zhao, Jie Zhong, Ping Lu, Xiao Feng, Ying Han, Chenqi Ling, Wenke Guo, Weijin Zhou, Fudong Yu

**Affiliations:** ^1^NHC Key Laboratory of Reproduction Regulation (Shanghai Institute of Planned Parenthood Research), Public Health School, Fudan University, Shanghai, China; ^2^Department of Gynecology, Shidong Hospital, Yangpu District, Shanghai, China; ^3^Department of Gynecology, Shanghai First Maternity and Infant Hospital, Tongji University School of Medicine, Shanghai, China

## Abstract

Ovarian carcinoma (OV) is a lethal gynecological malignancy. Most OV patients develop resistance to platinum-based chemotherapy and recurrence. Peroxisome proliferator-activated receptors (PPARs) are the ligand activating transcription factor of the nuclear receptor superfamily. PPARs as important transcriptional regulators regulate important physiological processes such as lipid metabolism, inflammation, and wound healing. Several reports point out that PPARs can also have an effect on the sensitivity of tumor cells to platinum-based chemotherapy drugs. However, the role of PPAR-target related genes (PPAR-TRGs) in chemotherapeutic resistance of OV remains unclear. The present study is aimed at optimizing candidate genes by integrating platinum-chemotherapy expression data and PPAR family genes with their targets. The gene expression profiles were obtained from Gene Expression Omnibus (GEO) and The Cancer Genome Atlas (TCGA) database. A total of 4 genes (*AP2A2*, *DOCK4*, *HSDL2*, and *PDK4*) were the candidate differentially expressed genes (DEGs) of PPAR-TRGs with platinum chemosensitivity. After conducting numerous survival analyses using different cohorts, we found that only the upexpression of *DOCK4* has important significance with the poor prognosis of OV patients. Meanwhile, *DOCK4* is detected in plasma and enriched in neutrophil and monocyte cells of the blood. We further found that there were significant correlations between *DOCK4* expression and the levels of CD4+ T cell infiltration, dendritic cell infiltration, and neutrophil infiltration in OV. In addition, we verified the expression level of *DOCK4* in OV cell lines treated with platinum drugs and found that *DOCK4* is potentially responsive to platinum drugs. In conclusion, *DOCK4* is potentially associated with immune cell infiltration and represents a valuable prognostic biomarker in ovarian cancer patients.

## 1. Introduction

Ovarian cancer (OV) is the fifth-leading cause of mortality among women with gynecological tumors in modern society. There are more than 240 000 women diagnosed with OV each year in the world. OV mainly comprises three large types, namely, epithelial, germ cell, and specialized stromal cell tumors, of which epithelial ovarian cancer is the most common type of ovarian cancer. Epithelial ovarian cancer can be further classified into five histological subtypes including high-grade serous, low-grade serous, endometriosis, clear cell, and mucinous ovarian carcinoma. Since 70% of advanced cancer patients are diagnosed at stage III or IV, they suffer from poor prognosis with tumor metastases, relapses, and even death from the disease [1, 2]. Nowadays, tumor debulking surgery followed by platinum-taxane chemotherapy is the primary treatment. However, the platinum-resistant cancer recurrence rate is close to 25% within six months [3, 4]. The lack of effective adjuvant therapeutics requires a greater understanding of the biology of its progression. Despite intensive research efforts, the overall survival (OS) of patients has been slightly improved over the past years. Reliable biomarkers, as a potential improvement for OV patients involving detection, diagnosis, prognosis, response to therapy, and outcome, are urgently required. Therefore, this article hopes to find out the prognostic biomarkers related to ovarian cancer chemotherapy sensitivity by studying the relationship between peroxisome proliferator-activated receptors (PPARs) and OV chemotherapy sensitivity.

PPARs are ligand-activated transcription factors which belong to the nuclear receptor superfamily including PPAR*α*, PPAR*β*/*δ*, and PPAR*γ* three isoforms [5, 6]. PPARs interact with other transcription regulators to regulate the transcription of its target genes involved in energy metabolism and important cellular biological functions like inflammation, cellular proliferation, and differentiation [7, 8]. In recent years, it has been reported that PPARs can also have an effect on the sensitivity of tumor cells to platinum-based chemotherapy drugs [9]. Increasing evidence shows that PPARs are important regulators of innate immunity and inflammatory response [10]. Given these crucial biological process regulatory roles of PPARs, abnormal expression of PPARs is associated with chronic diseases, such as diabetes, obesity, and cardiovascular disease [11]. Likewise, several previous studies suggested that PPAR involved processes were correlated with tumorigenesis including terminal differentiation, cell cycle arrest, and apoptosis of cancer cells [12]. In addition, the excessive activation of PPARs can lead to the increase of regulatory T cells and immunosuppression. However, the role of PPARs in ovarian cancer was poorly understood. Therefore, it will contribute to the prognosis to evaluate gene expression patterns in various cancers.

To further explore the roles of PPAR-target related genes (PPAR-TRGs) in OV prognosis, we collected GEO database (GSE51373, GSE63885) from primary patients that underwent chemotherapy to analyze the differences in chemotherapeutic sensitivity of platinum, then screened the candidate genes from the PPAR gene database, and finally used TCGA database for prognostic survival analysis; we found that *DOCK4* is the regulatory gene of PPARs which is associated with chemotherapy sensitivity and OV prognosis. This article will further analyze and study *DOCK4*, Rho GTPases, a receptor for calcium adhesions, which can drive the cytoskeleton reorganization, which is widely studied in cell adhesion and migration [13]. The *DOCK* family is a nonclassical type of cancer-associated Rho GTPase exchange factor. *DOCK4*, as a key guanine nucleotide exchange factor, is involved in regulation of the small GTPase Rac1 and a Ras-like small GTPase Rap1. It is increasingly recognized that *DOCK4* induced Rac activation and *Wnt/β*-catenin pathway to stimulate cell polarization, migration, and invasion, which are associated with cancer progression and metastasis. For instance, *DOCK4* forms a complex with *ELMO* and *SH3YL1* to induce Rac-dependent cell migration [14].

To investigate the deregulation of PPAR target gene *DOCK4* and involved mechanism in ovarian cancer, we use public data to analyze the characteristics of chemotherapy sensitivity and prognostic survival analysis and then found that there were significant correlations between *DOCK4* expression and the levels of CD4+ T cell infiltration, dendritic cell infiltration, and neutrophil infiltration in ovarian cancer. Meanwhile, *DOCK4* is detected in plasma and enriched in neutrophil and monocyte cells of the blood. The results identify that *DOCK4* is promising to become a prognostic biomarker related to OV chemotherapy.

## 2. Materials and Methods

### 2.1. Patient Information

#### 2.1.1. TCGA Cohort

The data used for our analysis based on datasets of The Cancer Genome Atlas Research Network (TCGA) is retrieved from UCSC (http://xena.ucsc.edu/public). In whole, we include a total of *N* = 304 ovarian cancer samples of RNA-Seq data (Illumina HiSeq pancan normalized data) with nonzero OS time from the latest TCGA sequencing sample. The clinical information of OV samples was revised by the TCGA Pan-Cancer Clinical Data Resource (TCGA-CDR) [15].

#### 2.1.2. Validation Cohort

Two independent cohorts (AOCS, MSKCC) are enrolled in this project as validation cohort datasets. The AOCS (Australian Ovarian Cancer Study) cohort (*n* = 278) samples (1992–2006) have more than 5 years of follow-up clinical information. The MSKCC cohort (*n* = 195) samples (1990–2003) have more than 5 years of follow-up. The mRNA expression data are as follows: AOCS cohort (GSE9891) and MSKCC cohort (GSE26172) using the Affymetrix human U133A microarray downloaded from GEO datasets.

#### 2.1.3. Platinum-Treated Patients

Information of patients receiving platinum chemotherapy is downloaded from GEO datasets (GSE51373 (*n* = 28) and GSE63885 (*n* = 75)). We divide the patients in each dataset into two groups: chemotherapy-sensitive (GSE51373—18 samples, GSE63885—41 samples) and chemotherapy-resistant (GSE51373—10 samples, GSE63885—34 samples) groups on the basis of their Platinum-Free Interval (PFI), defined as the time between the last dose of first-line carboplatin-based chemotherapy and the date of tumor progression; patients were defined as “resistant” (PFI < 6 months), “partially sensitive” (PFI 6-12 months), and “sensitive” (PFI > 12 months). “Partially sensitive” is included in the sensitive group [16].

### 2.2. Data of PPAR Family Genes and Their Targets

The candidate genes about the PPAR family transcription factors and their target genes are downloaded from the PPAR gene database [17]. The PPAR target genes used in this study are selected by those experimentally verified targets. After removing redundant genes, there were 130 genes involved in this study with the candidate genes PPAR-TRGs of the PPAR family transcription factors and target genes.

### 2.3. Platinum-Treated Cell Lines

To investigate the cellular responses of ovarian cancer cells to cisplatin, we performed transcriptome analysis in 46 ovarian cancer cell lines treated with GI50 doses of cisplatin (data from GSE47856). (GI50 is the cisplatin dosage required to cause a 50% reduction in the increase in viable cell number over 48 h as compared with untreated control cells).

### 2.4. Immune Cell Infiltration Analysis

TIMER (https://cistrome.shinyapps.io/timer/) is a database designed for analyzing immune cell infiltration in multiple cancers [18]. This database employs pathological examination-validated statistical methodology in order to estimate tumor immune infiltration by neutrophils, macrophages, dendritic cells, B cells, and CD4/CD8 T cells. We further executed Kaplan-Meier curve analyses to explore the survival of PPAR-TRG candidates and immune cells. In addition, this webserver could analyze the correlation relationship between gene expression and immune cells.

### 2.5. *DOCK4* Prognosis Analysis

The Kaplan-Meier plotter offers a means of readily exploring the impact of a wide array of genes on patient survival in 21 different types of cancer, with large sample sizes for the ovarian (*n* = 2190) cancer cohorts [19]. We therefore used this database to explore the association between *DOCK4* expression and outcome in patients with ovarian cancer. The candidate gene survival analysis used R packages “survival,” “survminer,” “glmnet,” and “dplyr.” The cutoff of *p* value was 0.05 by using log-rank *p* value.

### 2.6. Statistical Analysis

Kaplan-Meier plotter and TIMER databases were used for generating survival plots in respective analysis, with data including either HR and *p* values or *p* values derived from a log-rank test. Statistical analyses were performed using R software v3.5.0 and GraphPad Prism v5.00. We used the multivariable Cox proportional hazard model to analyze prognosis-related multivariate of ovarian cancer. We used a limma package to analyze the mRNA gene expression for different expression genes. The selection of cutoff was 0.05 in this study. In addition, we used R packages “survival,” “survminer,” “glmnet,” and “dplyr” to produce survival plots.

## 3. Results

### 3.1. Optimization of Candidate Genes of PPAR Family Genes with Their Targets by Integrating Platinum-Chemotherapy Expression Data and Survival Signature

**Figure 1:**
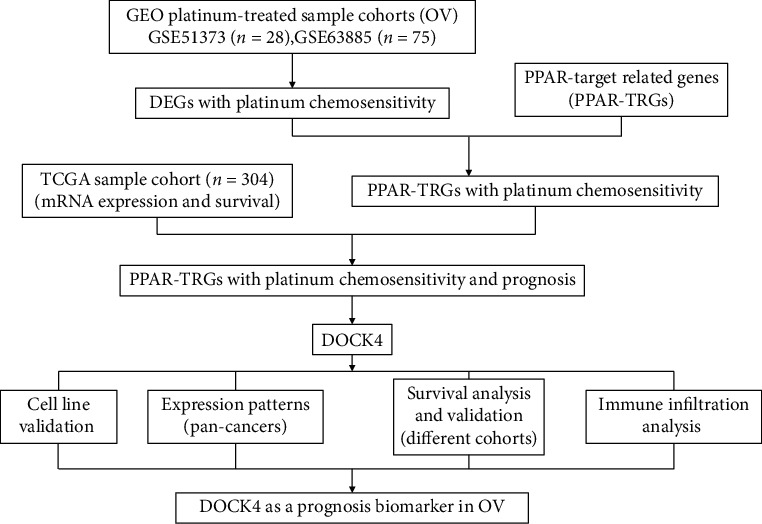
The flow chart shows the scheme of our study on mRNA prognostic signatures for OV (ovarian cancer).

For the sake of obtaining candidate genes related to PPARs and platinum chemosensitivity, we collected platinum-chemotherapy expression data of OV in GEO database (GSE51373 (*n* = 28) and GSE63885 (*n* = 75)) and divided each dataset into the chemotherapy-sensitive (GSE51373—18 samples, GSE63885—41 samples) and chemotherapy-resistant (GSE51373—10 samples, GSE63885—34 samples) groups. Compared with the sensitive group, 269 DEGs were platinum sensitive in OV. It is increasingly recognized that PPARs as transcription regulators play critical roles in a great amount of cellular function. Based on previous studies, we collected 143 candidate gene PPAR-TRGs from the PPAR gene database (http://www.ppargene.org/.). There were only 4 PPAR-TRGs (*AP2A2*, *DOCK4*, *HSDL2*, and *PDK4*) out of 269 DEGs, which are associated with platinum chemosensitivity in ovarian cancer. Subsequently, we analyzed the survival analysis of these 4 genes by using the clinical information of RNA-Seq ovarian cancer patients from TCGA database. Finally, we found that only the upexpression of *DOCK4* has significant clinical outcome with the poor prognosis of OV patients. We conducted our study as described in the flow chart (Figure 1). Therefore, we have reason to believe that *DOCK4* might be the potential prognosis biomarker for ovarian cancer.

### 3.2. Overexpression of *DOCK4* Predicts Poor Prognosis for Ovarian Cancer

**Figure 2:**
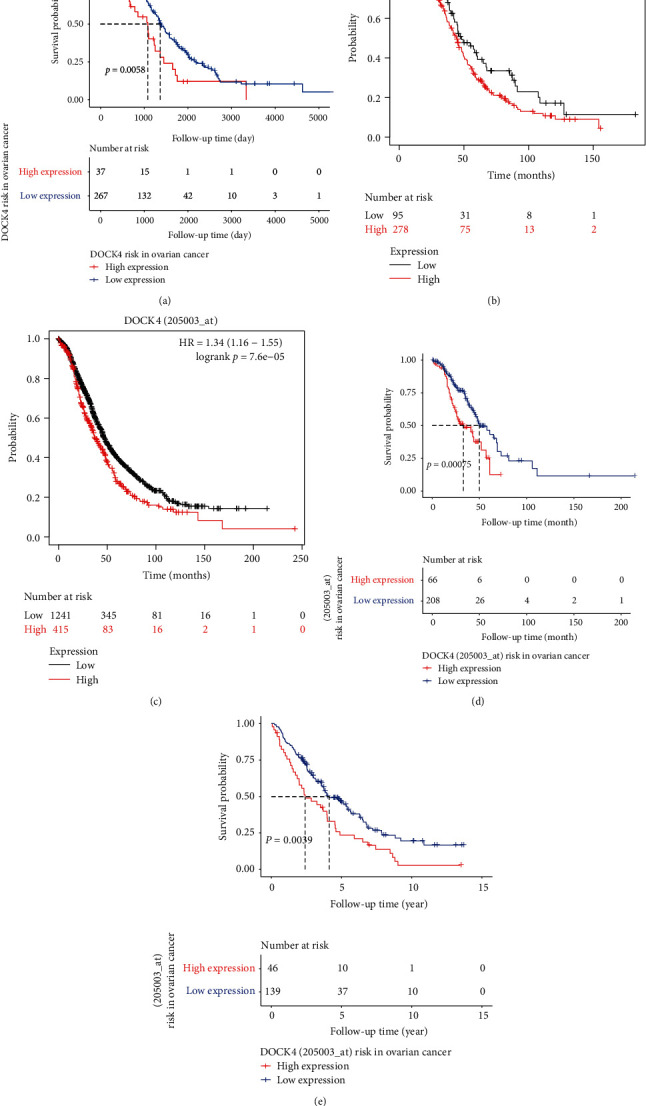
The prognostic value of DOCK4 in OV. Kaplan-Meier curves show the association between DOCK4 expression and overall survival, using data from (a) TCGA, (b, c) Kaplan-Meier plotter, and (d, e) GEO in ovarian cancer.

In order to decipher the prognostic value of *DOCK4* in patients with OV, we explored the link between the expression of *DOCK4* and clinical outcome from TCGA patient dataset with only RNA-Seq expression data (*n* = 304) (Figure 2(a)). We found that *DOCK4* upexpression was associated with a worse prognosis in ovarian cancer. To verify DOCK4 clinical significance in ovarian cancer, we used other datasets such as array expression data (*n* = 1656) and RNA-Seq expression data (*n* = 374) by using the webserver of Kaplan-Meier plotter (https://kmplot.com/analysis/). The overall survival analysis results of array data (Figure 2(b)) and RNA-Seq data (Figure 2(c)) from Kaplan-Meier plotter indicated that DOCK4 gene upexpression was a poor prognosis of ovarian cancer patients. The analysis results from different data indicate that downexpression of *DOCK4* correlates with better clinical outcome. For further validation, we employed other two independent cohorts (AOCS (1992–2006), MSKCC (1990–2003)) from the GEO database to assess how *DOCK4* expression relates to prognosis in OV, revealing its elevation to be significantly linked with a poorer prognosis in OV (Figures 2(d) and 2(e)). In general, the upexpression of *DOCK4* is correlated with OS in OV patients. These results thus clearly demonstrate that *DOCK4* expression significantly correlated with poorer outcome in ovarian cancer and might be a potential prognostic biomarker for OV.

### 3.3. Assessment of *DOCK4* Expression Pattern in Pan-Cancers

**Figure 3:**
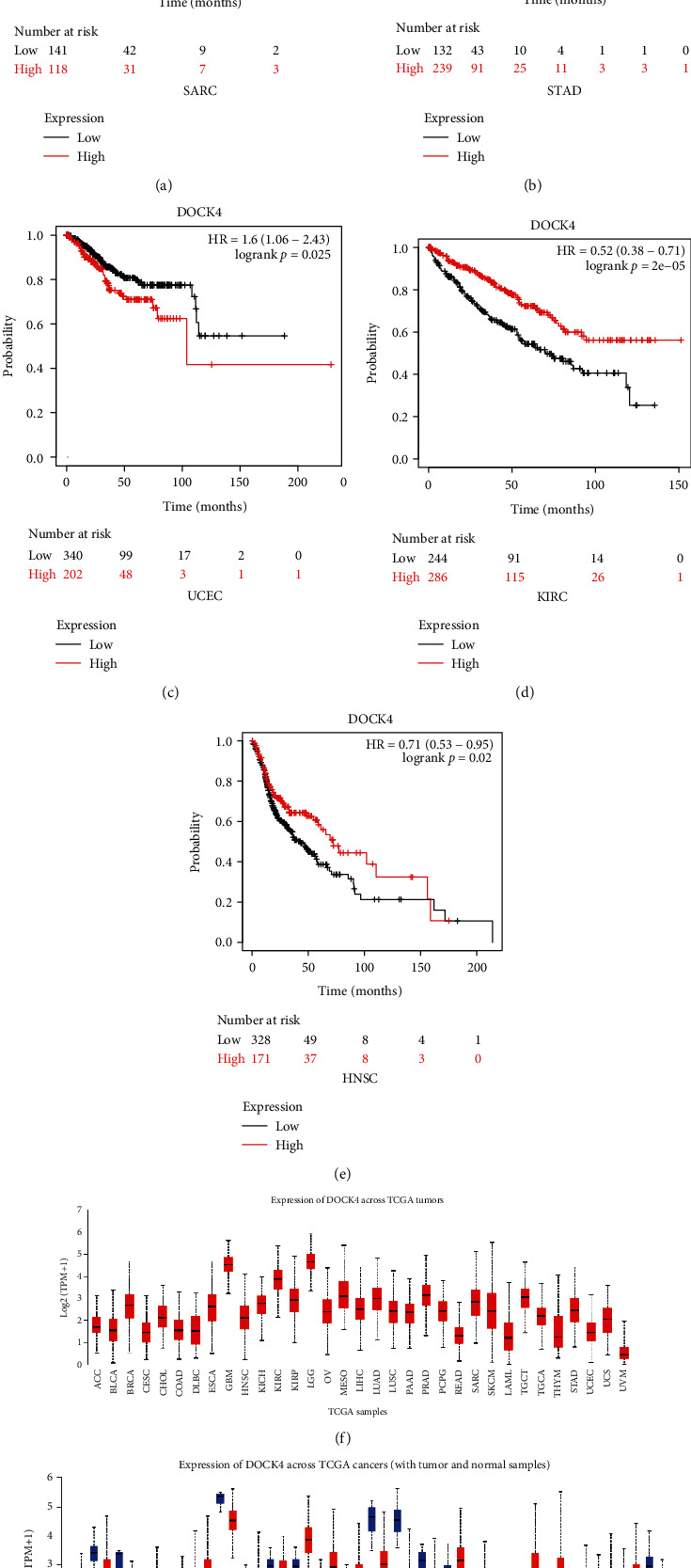
The survival analysis of DOCK4 in various cancers and expression patterns of DOCK4 across TCGA cancer types. (a–e) Kaplan-Meier curves show the correlation between DOCK4 expression and overall survival of SARC, STAD, UCEC, KIRC, and HNSC patients, respectively. (f) The summary of the expression pattern of DOCK4 across 33 tumors. ACC: adrenocortical carcinoma; BLCA: bladder urothelial carcinoma; BRCA: breast invasive carcinoma; CESC: cervical squamous cell carcinoma and endocervical adenocarcinoma; CHOL: cholangiocarcinoma; COAD: colon adenocarcinoma; DLBC: lymphoid neoplasm diffuse large B cell lymphoma; ESCA: esophageal carcinoma; GBM: glioblastoma multiforme; HNSC: head and neck squamous cell carcinoma; KICH: kidney chromophobe; KIRC: kidney renal clear cell carcinoma; KIRP: kidney renal papillary cell carcinoma; LGG: brain lower grade glioma; OV: ovarian cancer; MESO: mesothelioma; LIHC: liver hepatocellular carcinoma; LUAD: lung adenocarcinoma; LUSC: lung squamous cell carcinoma; PAAD: pancreatic adenocarcinoma; PCPG: pheochromocytoma and paraganglioma; PRAD: prostate adenocarcinoma; READ: rectum adenocarcinoma; SARC: sarcoma; SKCM: skin cutaneous melanoma; LAML: acute myeloid leukemia; TGCT: testicular germ cell tumors; THCA: thyroid carcinoma; THYM: thymoma; STAD: stomach adenocarcinoma; UCEC: uterine corpus endometrial carcinoma; UCS: uterine carcinosarcoma; UVM: uveal melanoma. (g) The changed expression of DOCK4 in 24 cancer cohorts compared to normal tissues.

To evaluate the possibility of *DOCK4* as a prognostic marker in different tumors, we applied different cancer tissue RNA-Seq datasets from TCGA datasets. The webserver of Kaplan-Meier plotter (https://kmplot.com/analysis/) was applied to do overall survival analysis by using RNA-Seq expression data of 33 tumors. The survival analysis result suggested high-expression DOCK4 gene as a potential poor prognosis biomarker among sarcoma (SARC), stomach adenocarcinoma (STAD), and uterine corpus endometrial carcinoma (UCEC) (Figures 3(a)–3(c)), while low-expression DOCK4 gene as a potential poor prognosis biomarker among kidney renal clear cell carcinoma (KIRC) and head and neck squamous cell carcinoma (HNSC) (Figures 3(d) and 3(e)). In order to detect DOCK4 expression pattern among different tumors, the webserver of UALCAN (http://ualcan.path.uab.edu/analysis.html) was employed to present DOCK4 gene expression pattern among 33 tumors with TCGA RNA-Seq expression data (Figure 3(f)), which indicated that DOCK4 gene does not have low expression in ovarian cancer. Furthermore, compared with normal tissues, *DOCK4* was relatively upexpressed in many cancer types including ESCA, HNSC, KIRC, KIRP, LIHC, PPAD, PCPG, SKCM, and STAD, while it is downexpressed in BLCA, BRCA, CESC, CHOL, COAD, GBM, KICH, LUAD, LUSC, PRAD, READ, SARC, THCA, THYM, and UCEC (Figure 3(g)). In summary, *DOCK4* has the potential to become a general biomarker in many tumors.

### 3.4. *DOCK4* Expression in Plasma

**Figure 4:**
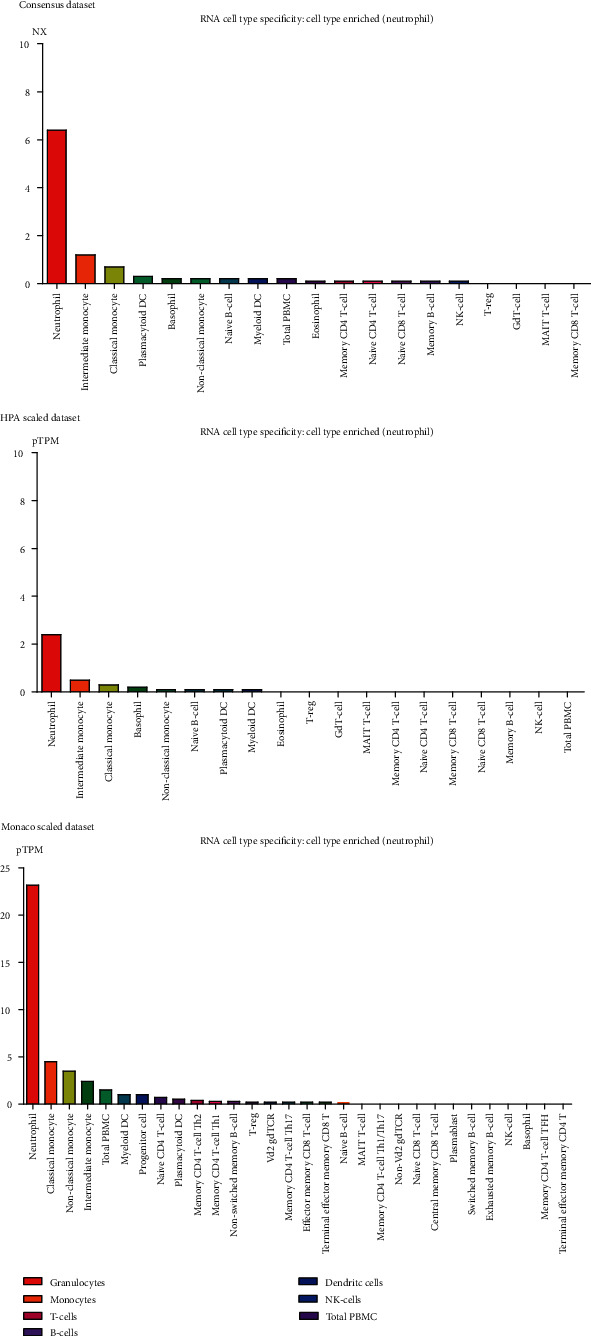
The relative expression of DOCK4 in various cells in peripheral blood.

To further explore the future application of *DOCK4* as a prognostic indicator to the clinic, in view of the previously known fact that *DOCK4* is highly expressed in OV tissues, we further tested the expression level of *DOCK4* in the blood and found that *DOCK4* can be detected as a secreted protein in peripheral blood in different datasets. In addition, *DOCK4* is specifically enriched in neutrophils (Figure 4), which indicated that DOCK4 could be a secreted protein detected in peripheral blood.

### 3.5. *DOCK4* Expression Correlates with Immune Cell Infiltration

**Figure 5:**
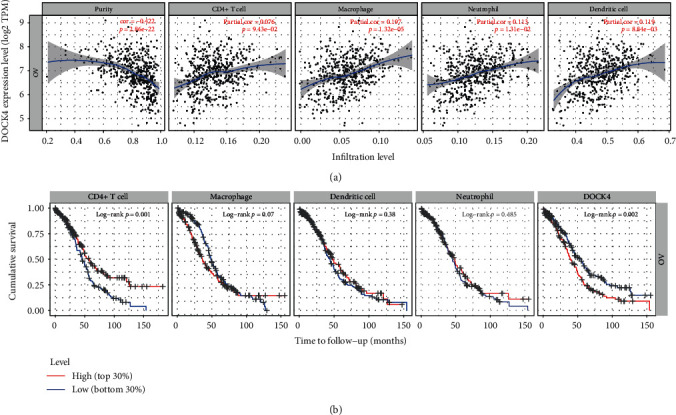
Assessment of the correlation between DOCK4 expression and immune cell infiltration. (a) DOCK4 expression is correlated with the level of immune infiltration in ovarian cancer. (b) Kaplan-Meier plots of immune infiltration and DOCK4 expression levels in ovarian cancer.

Since *DOCK4* is mainly enriched in neutrophils, it is necessary for us to study its relationship with other immune cells. Therefore, we analyzed the relationship between *DOCK4* expression and the degree of immune cell infiltration OV in the TIMER database. In 6 types of immune cells including B cells, CD4+ T cells, CD8+ T cells, neutrophils, macrophages, and dendritic cells, we found that *DOCK4* expression weakly positively correlated with the levels of CD4+ T cell infiltration, dendritic cell infiltration, and neutrophil infiltration in OV (Figure 5(a)). We further found CD4+ T cell infiltration to be significantly associated with OV prognosis (Figure 5(b)).

We explored the OV prognosis relevance of tumor immune subsets, with multiple covariates including age, ethnicity, *DOCK4* expression, and tumor stages in a multivariable Cox proportional hazard model. We found that only variables including age, CD4+ T cell, dendritic cell, neutrophils, macrophage, and *DOCK4* can be included in the model (Table 1).

This suggests that *DOCK4* plays a moderate role in interacting with immune cell infiltration in ovarian cancer. Certainly, further work will be necessary to identify the role of *DOCK4* in immune activity regulation in ovarian cancer.

### 3.6. *DOCK4* Expression in Ovarian Cell Lines with Platinum Treatment

**Figure 6:**
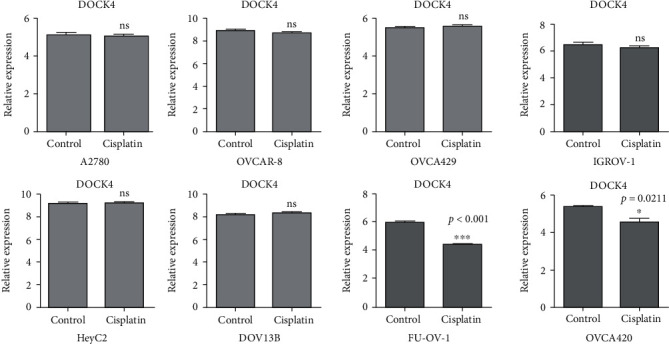
Changes in DOCK4 expression levels in some ovarian cancer cells treated with cisplatin.

We further want to verify the reactivity of *DOCK4* to platinum treatment in vitro. 46 ovarian cancer cell lines were treated with cisplatin (data from GSE47856), and we observed the changes in *DOCK4* expression levels. We found that *DOCK4* expression in OVCA420 and FU-OV-1 cell lines was significantly inhibited in the cisplatin-treated group compared with the control group (Figure 6). *DOCK4* expression in other cell lines was not significantly different which might be due to the dissimilarity between cell lines in vitro and tumor in vivo (S1). Therefore, to some extent, we believe that *DOCK4* might be sensitive to the treatment of platinum drugs and can be used as a prognostic indicator for certain types of OV selection of platinum chemotherapy drugs.

## 4. Discussion

PPARs as ligand-activated transcriptional factors play dominative biological functions such as glucose and lipid metabolism. However, PPAR-TRGs involved in platinum chemosensitivity and prognosis remain to be interpreted further. Ovarian cancer is the fifth common cancer accompanied with poor prognosis of which the 5-year relative survival is less than 45% [20]. Consequently, constructing molecular signatures of prognosis shows high priority to improve the treatment of these patients. This study mainly focused on illuminating the role of PPAR-TRGs in platinum chemosensitivity and prognosis of ovarian cancer to establish the molecular signature for clinical application. In this study, 4 DEGs were identified as the candidate genes of PPAR-TRGs with platinum chemosensitivity by analyzing chemotherapy-sensitive and chemotherapy-resistant samples. Meanwhile, our results suggest that *DOCK4*, a Rho GTPase exchange factor for Rac, is negatively correlated with the survival of ovarian cancer patients, supporting previous reports that the abnormal expression of *DOCK4* has been associated with tumor migration and metastasis in breast cancer and lung adenocarcinoma [21, 22]. Since *DOCK4* as a member of the *DOCK* family is targeted by PPARs which shows close association with numerous cancer types, we characterized the expression of *DOCK4* in 24 cancers. The result shows that *DOCK4* is upexpressed in 9 cancer species and downexpressed in 15 cancer species which may be due to the distribution and function of *DOCK* family 11 members which are variant in different tissues and cells. These results indicate that *DOCK4* might be a general biomarker for many cancer types. Further survival analysis demonstrated that *DOCK4* indeed correlates with prognosis of some cancer types. Therefore, much more efforts are required to clarify the relationship between the *DOCK* family and the cancer process.

Given that *DOCK4*, the promising biomarker for prognosis of ovarian cancer patients, could be detected in blood contributing to clinical treatment, we then investigated the expression pattern of *DOCK4* in blood samples consistent with *DOCK4* encoding a secreted protein. Our results showed that *DOCK4* could be detected in the peripheral blood of ovarian cancer patients. It is widely recognized that the infiltration of tumor-infiltrating lymphocytes (TILs) has been positively associated with prognosis and platinum chemosensitivity in a great deal of cancers, including ovarian cancer [23–25]. In parallel with the importance of immune response in cancers, our results further confirmed that *DOCK4* was mainly enriched in neutrophils. Several reports have confirmed that neutrophils participate in the regulation of inflammation and the induction of angiogenesis within the tumor. On the other hand, numerous studies have indicated that PPARs play important roles in innate immunity and inflammatory response which are involved in tumor progression and cancer cell metabolism. In particular, PPAR*β*/*δ* also has an effect on proangiogenic effects in several researches [26]. Therefore, the role of *DOCK4* in neutrophils remains to be explored in ovarian cancer progression. We further found that the expression of *DOCK4* is correlated with the levels of CD4+ T cell infiltration, dendritic cell infiltration, and neutrophil infiltration in these ovarian cancer patients. Combined with previous studies, our results demonstrated that *DOCK4* targeted by PPAR*δ* has a hand in immunological construction. Therefore, much more efforts are demanded to reveal the mechanism of *DOCK4* targeted by PPAR*δ* in ovarian cancer patients' immune cells, especially neutrophils.

Since ovarian cancer is a heterogeneous disease encompassing a group of neoplasms with distinct clinicopathological and genetic features [27], the cancer sample size should be enlarged and classified into different groups for extended research. Furthermore, other experimental methods in vivo and in vitro would be considered to characterize the regulation mechanism of *DOCK4* in ovarian cancer. Other members of the *DOCK* family might be further explored to contribute to the related research of ovarian cancer.

## 5. Conclusion

In this study, we firstly identified a potential function suggesting the chemotherapy-sensitive role of *DOCK4* in ovarian cancer, which might be correlated with immune cell infiltration. Altogether, *DOCK4* might be a candidate prognosis biomarker for ovarian cancer patients. The function and mechanism of *DOCK4* in ovarian cancer need further research.

## Figures and Tables

**Table 1:** Multivariate analysis associated with overall survival in ovarian cancer patients (*n* = 547).

Parameters	Coefficient	HR (95% CI)	*p*
Age	0.022	1.022 (1.012-1.033)	0.000
CD4+ T cell	-11.633	0.000 (0.000-0.004)	0.000
Macrophage	5.307	201.833 (0.897-45431.169)	0.055
Dendritic cell	-3.703	0.025 (0.001-0.947)	0.047
Neutrophil	9.762	17362.900 (4.858-62051075.704)	0.019
DOCK4	0.248	1.281 (1.092-1.503)	0.002

## Data Availability

The data in our study are available from the corresponding author upon reasonable request.

## Supplementary Materials

Supplementary Materials File 1: changes in DOCK4 expression levels in all cell lines treated with cisplatin.
