# Supplementary material for: DOCK4 Is a Platinum-Chemosensitive and Prognostic-Related Biomarker in Ovarian Cancer
Source: PPAR Res. 2021 Feb 3;2021:6629842. doi: 10.1155/2021/6629842 (PMC7878079; doi:10.1155/2021/6629842)
Supplement: Supplementary Materials — File 1: changes in DOCK4 expression levels in all cell lines treated with cisplatin. [file 6629842.f1.pdf]

0	#Sample_t	#Sample_characteristics_ch1	#Sample_c	#"ID_REF"	DOCK4
1	HeyA8-(1)	cell line: HeyA8	treatment:	GSM11607	9.42021
2	HeyA8-(1)	cell line: HeyA8	treatment:	GSM11607	9.50655
3	HeyA8-(2)	cell line: HeyA8	treatment:	GSM11607	9.37168
4	HeyA8-(2)	cell line: HeyA8	treatment:	GSM11607	9.19044
5	HeyA8-(3)	cell line: HeyA8	treatment:	GSM11607	9.48152
6	HeyA8+(1)	cell line: HeyA8	treatment:	GSM11607	9.44367
7	HeyA8+(3)	cell line: HeyA8	treatment:	GSM11607	9.44024
8	HeyC2-(1)	cell line: HeyC2	treatment:	GSM11607	9.16726
9	HeyC2-(2)	cell line: HeyC2	treatment:	GSM11607	9.03573
10	HeyC2-(3)	cell line: HeyC2	treatment:	GSM11607	9.14084
11	HeyC2+(1)	cell line: HeyC2	treatment:	GSM11607	9.30026
12	HeyC2+(2)	cell line: HeyC2	treatment:	GSM11607	9.12603
13	HeyC2+(3)	cell line: HeyC2	treatment:	GSM11607	9.13409
14	A2780-(1)	cell line: A2780	treatment:	GSM11607	4.84595
15	A2780-(2)	cell line: A2780	treatment:	GSM11607	5.21821
16	A2780-(3)	cell line: A2780	treatment:	GSM11607	5.30618
17	A2780+(1)	cell line: A2780	treatment:	GSM11607	5.2474
18	A2780+(2)	cell line: A2780	treatment:	GSM11607	4.89317
19	A2780+(3)	cell line: A2780	treatment:	GSM11607	5.07674
20	OVCA420-	cell line: OVCA420	treatment:	GSM11607	5.50973
21	OVCA420-	cell line: OVCA420	treatment:	GSM11607	5.25356
22	OVCA420-	cell line: OVCA420	treatment:	GSM11607	5.36976
23	OVCA420-	cell line: OVCA420	treatment:	GSM11607	4.42978
24	OVCA420-	cell line: OVCA420	treatment:	GSM11607	4.27653
25	OVCA420-	cell line: OVCA420	treatment:	GSM11607	4.96762
26	OVCA429-	cell line: OVCA429	treatment:	GSM11607	5.40756
27	OVCA429-	cell line: OVCA429	treatment:	GSM11607	5.44792
28	OVCA429-	cell line: OVCA429	treatment:	GSM11607	5.66484
29	OVCA429-	cell line: OVCA429	treatment:	GSM11607	5.74809
30	OVCA429-	cell line: OVCA429	treatment:	GSM11607	5.48539
31	OVCA429-	cell line: OVCA429	treatment:	GSM11607	5.54329
32	PA-1-(1)	cell line: PA-1	treatment:	GSM11607	7.95037
33	PA-1-(2)	cell line: PA-1	treatment:	GSM11607	7.78515
34	PA-1-(3)	cell line: PA-1	treatment:	GSM11607	7.71464
35	PA-1+(1)	cell line: PA-1	treatment:	GSM11607	8.07234
36	PA-1+(2)	cell line: PA-1	treatment:	GSM11607	8.03653
37	PA-1+(3)	cell line: PA-1	treatment:	GSM11607	7.99675
38	TYK-nu-(1	cell line: TYK-nu	treatment:	GSM11607	9.47425
39	TYK-nu-(2	cell line: TYK-nu	treatment:	GSM11607	9.37168
40	TYK-nu-(3	cell line: TYK-nu	treatment:	GSM11607	9.45514
41	TYK-nu+(1	cell line: TYK-nu	treatment:	GSM11607	9.10242
42	TYK-nu+(2	cell line: TYK-nu	treatment:	GSM11607	9.01392
43	TYK-nu+(3	cell line: TYK-nu	treatment:	GSM11607	8.98587
44	CH1-(1)	cell line: CH1	treatment:	GSM11607	7.3094
45	CH1-(2)	cell line: CH1	treatment:	GSM11607	7.42787
46	CH1-(3)	cell line: CH1	treatment:	GSM11607	7.44942
47	CH1+(1)	cell line: CH1	treatment:	GSM11607	7.87007
48	CH1+(2)	cell line: CH1	treatment:	GSM11607	7.63187
49	CH1+(3)	cell line: CH1	treatment:	GSM11607	7.63631
50	FU-OV-1-	cell line: FU-OV-1	treatment:	GSM11607	5.93619
51	FU-OV-1-	cell line: FU-OV-1	treatment:	GSM11607	5.89968
52	FU-OV-1-	cell line: FU-OV-1	treatment:	GSM11607	5.99979
53	FU-OV-1+	cell line: FU-OV-1	treatment:	GSM11607	4.35102
54	FU-OV-1+	cell line: FU-OV-1	treatment:	GSM11607	4.38147
55	FU-OV-1+	cell line: FU-OV-1	treatment:	GSM11607	4.42378
56	A2008-(1)	cell line: A2008	treatment:	GSM11607	4.76097
57	A2008-(2)	cell line: A2008	treatment:	GSM11607	4.6288

58	A2008-(3)	cell line: A2008	treatment:	GSM11607	6.96578
59	A2008+(1)	cell line: A2008	treatment:	GSM11607	7.35922
60	A2008+(2)	cell line: A2008	treatment:	GSM11607	4.87808
61	A2008+(3)	cell line: A2008	treatment:	GSM11607	5.13482
62	DOV13-(1	cell line: DOV13	treatment:	GSM11607	7.84088
63	DOV13-(1	cell line: DOV13	treatment:	GSM11607	7.98231
64	DOV13-(2	cell line: DOV13	treatment:	GSM11607	7.91947
65	DOV13-(2	cell line: DOV13	treatment:	GSM11607	7.8822
66	DOV13-(3	cell line: DOV13	treatment:	GSM11607	7.82123
67	DOV13+(1	cell line: DOV13	treatment:	GSM11607	8.35958
68	DOV13+(2	cell line: DOV13	treatment:	GSM11607	8.33155
69	DOV13+(3	cell line: DOV13	treatment:	GSM11607	8.37606
70	OVCA433-	cell line: OVCA433	treatment:	GSM11607	5.13857
71	OVCA433-	cell line: OVCA433	treatment:	GSM11607	4.90057
72	OVCA433-	cell line: OVCA433	treatment:	GSM11608	4.93059
73	OVCA433-	cell line: OVCA433	treatment:	GSM11608	5.054
74	OVCA433-	cell line: OVCA433	treatment:	GSM11608	5.20006
75	OVCA433-	cell line: OVCA433	treatment:	GSM11608	5.22705
76	OVCAR-10	cell line: OVCAR-10	treatment:	GSM11608	5.41304
77	OVCAR-10	cell line: OVCAR-10	treatment:	GSM11608	5.41949
78	OVCAR-10	cell line: OVCAR-10	treatment:	GSM11608	5.58265
79	OVCAR-10	cell line: OVCAR-10	treatment:	GSM11608	7.73416
80	OVCAR-10	cell line: OVCAR-10	treatment:	GSM11608	5.23139
81	OVCAR-10	cell line: OVCAR-10	treatment:	GSM11608	4.99041
82	DOV13B-(	cell line: DOV13B	treatment:	GSM11608	8.06575
83	DOV13B-(	cell line: DOV13B	treatment:	GSM11608	8.1313
84	DOV13B-(	cell line: DOV13B	treatment:	GSM11608	8.16154
85	DOV13B+(	cell line: DOV13B	treatment:	GSM11608	8.17464
86	DOV13B+(	cell line: DOV13B	treatment:	GSM11608	8.31926
87	DOV13B+(	cell line: DOV13B	treatment:	GSM11608	8.41892
88	C13-(1)	cell line: C13	treatment:	GSM11608	4.3119
89	C13-(2)	cell line: C13	treatment:	GSM11608	4.28845
90	C13-(3)	cell line: C13	treatment:	GSM11608	4.22166
91	C13+(1)	cell line: C13	treatment:	GSM11608	4.25848
92	C13+(2)	cell line: C13	treatment:	GSM11608	4.25732
93	C13+(3)	cell line: C13	treatment:	GSM11608	4.06864
94	IGROV-1-	cell line: IGROV-1	treatment:	GSM11608	6.63118
95	IGROV-1-	cell line: IGROV-1	treatment:	GSM11608	6.67254
96	IGROV-1-	cell line: IGROV-1	treatment:	GSM11608	6.09884
97	IGROV-1+	cell line: IGROV-1	treatment:	GSM11608	5.93103
98	IGROV-1+	cell line: IGROV-1	treatment:	GSM11608	6.41637
99	IGROV-1+	cell line: IGROV-1	treatment:	GSM11608	6.31889
100	OVCAR-8-	cell line: OVCAR-8	treatment:	GSM11608	9.10866
101	OVCAR-8-	cell line: OVCAR-8	treatment:	GSM11608	8.86119
102	OVCAR-8-	cell line: OVCAR-8	treatment:	GSM11608	8.8482
103	OVCAR-8-	cell line: OVCAR-8	treatment:	GSM11608	8.85494
104	OVCAR-8-	cell line: OVCAR-8	treatment:	GSM11608	8.68958
105	OVCAR-8-	cell line: OVCAR-8	treatment:	GSM11608	8.64719
106	M41(-)	cell line: M41	treatment:	GSM11608	3.19611
107	M41(+)	cell line: M41	treatment:	GSM11608	2.9143
108	A2780cisR	cell line: A2780cisR	treatment:	GSM11608	4.12148
109	A2780cisR	cell line: A2780cisR	treatment:	GSM11608	3.66482
110	ovary1847	cell line: ovary1847	treatment:	GSM11608	4.68887
111	ovary1847	cell line: ovary1847	treatment:	GSM11608	5.15037
112	Caov-2(-)	cell line: Caov-2	treatment:	GSM11608	7.43823
113	Caov-2(+)	cell line: Caov-2	treatment:	GSM11608	7.18057
114	Caov-3(-)	cell line: Caov-3	treatment:	GSM11608	5.95632
115	Caov-3(+)	cell line: Caov-3	treatment:	GSM11608	6.00361

116	Hey(-)	cell line: Hey	treatment: GSM11608	8.23032
117	Hey(+)	cell line: Hey	treatment: GSM11608	6.79473
118	JHOS-2(-)	cell line: JHOS-2	treatment: GSM11608	5.44056
119	JHOS-2(+)	cell line: JHOS-2	treatment: GSM11608	4.9073
120	JHOS-3(-)	cell line: JHOS-3	treatment: GSM11608	6.01246
121	JHOS-3(+)	cell line: JHOS-3	treatment: GSM11608	5.91583
122	OAW28(-)	cell line: OAW28	treatment: GSM11608	4.16348
123	OAW28(+)	cell line: OAW28	treatment: GSM11608	3.61017
124	OAW42(-)	cell line: OAW42	treatment: GSM11608	5.33339
125	OAW42(+)	cell line: OAW42	treatment: GSM11608	5.2457
126	DOV13A(-	cell line: DOV13A	treatment: GSM11608	7.22487
127	DOV13A(+	cell line: DOV13A	treatment: GSM11608	7.74853
128	OV56(-)	cell line: OV56	treatment: GSM11608	7.12932
129	OV56(+)	cell line: OV56	treatment: GSM11608	4.27211
130	OVCAR-3(	cell line: OVCAR-3	treatment: GSM11608	6.87951
131	OVCA432(	cell line: OVCA432	treatment: GSM11608	3.33249
132	OVCA432(	cell line: OVCA432	treatment: GSM11608	3.40211
133	OVCA433b	cell line: OVCA433	treatment: GSM11608	3.74685
134	OVCA433b	cell line: OVCA433	treatment: GSM11608	3.83908
135	OVCAR-2(	cell line: OVCAR-2	treatment: GSM11608	7.32807
136	OVCAR-2(	cell line: OVCAR-2	treatment: GSM11608	6.03575
137	OVCAR-3(	cell line: OVCAR-3	treatment: GSM11608	7.08737
138	OVCAR-5(	cell line: OVCAR-5	treatment: GSM11608	7.63407
139	OVCAR-5(	cell line: OVCAR-5	treatment: GSM11608	7.3376
140	OVK-18(-)	cell line: OVK-18	treatment: GSM11608	6.50849
141	OVK-18(+	cell line: OVK-18	treatment: GSM11608	6.30529
142	PA-1(-)	cell line: PA-1	treatment: GSM11608	6.8707
143	PEO1(-)	cell line: PEO1	treatment: GSM11608	4.70323
144	PEO1(+)	cell line: PEO1	treatment: GSM11608	4.51247
145	RMG-I(-)	cell line: RMG-I	treatment: GSM11608	6.35141
146	RMG-I(+)	cell line: RMG-I	treatment: GSM11608	5.33643
147	RMG-II(-)	cell line: RMG-II	treatment: GSM11608	5.40058
148	RMG-II(+)	cell line: RMG-II	treatment: GSM11608	5.0121
149	SKOV-4(-)	cell line: SKOV-4	treatment: GSM11608	7.27884
150	SKOV-4(+	cell line: SKOV-4	treatment: GSM11608	7.25487
151	SKOV-3-(1	cell line: SKOV-3	treatment: GSM11608	5.23105
152	SKOV-3-(2	cell line: SKOV-3	treatment: GSM11608	5.05846
153	SKOV-3(+	cell line: SKOV-3	treatment: GSM11608	4.63122
154	SKOV-6(-)	cell line: SKOV-6	treatment: GSM11608	6.36181
155	SKOV-6(+	cell line: SKOV-6	treatment: GSM11608	7.40102
156	SKOV-8(-)	cell line: SKOV-8	treatment: GSM11608	8.34588
157	SKOV-8(+	cell line: SKOV-8	treatment: GSM11608	7.75115
158	TAYA(-)	cell line: TAYA	treatment: GSM11608	5.58204
159	TAYA(+)	cell line: TAYA	treatment: GSM11608	4.95084
160	TOV-112D	cell line: TOV-112D	treatment: GSM11608	5.7184
161	TOV-112D	cell line: TOV-112D	treatment: GSM11608	5.53563
162	TOV-21G(	cell line: TOV-21G	treatment: GSM11608	5.62837
163	TOV-21G(	cell line: TOV-21G	treatment: GSM11608	6.17054
164	UWB1.289	cell line: UWB1.289	treatment: GSM11608	7.12715
165	UWB1.289	cell line: UWB1.289	treatment: GSM11608	6.21008
